# Virtual Reality Relaxation to Decrease Dental Anxiety: Immediate Effect Randomized Clinical Trial

**DOI:** 10.1177/2380084420901679

**Published:** 2020-01-21

**Authors:** S. Lahti, A. Suominen, R. Freeman, T. Lähteenoja, G. Humphris

**Affiliations:** 1 Department of Community Dentistry, University of Turku, Turku, Finland; 2 Turku Clinical Research Centre, Turku University Hospital, Turku, Finland; 3 Dental Health Services Research Unit, School of Dentistry, University of Dundee, Dundee, UK; 4 Division of Populations and Behavioural Science, School of Medicine, University of St Andrews, St Andrews, UK

**Keywords:** dental fear, clinical studies/trials, relaxation technics, virtual reality immersion, dental care, public sector

## Abstract

**Introduction:**

Dental anxiety is common and causes symptomatic use of oral health services.

**Objectives:**

The aim was to study if a short-term virtual reality intervention reduced preoperative dental anxiety.

**Methods:**

A randomized controlled single-center trial was conducted with 2 parallel arms in a public oral health care unit: virtual reality relaxation (VRR) and treatment as usual (TAU). The VRR group received a 1- to 3.5-min 360° immersion video of a peaceful virtual landscape with audio features and sound supporting the experience. TAU groups remained seated for 3 min. Of the powered sample of 280 participants, 255 consented and had complete data. Total and secondary sex-specific mixed effects linear regression models were completed for posttest dental anxiety (Modified Dental Anxiety Scale [MDAS] total score) and its 2 factors (anticipatory and treatment-related dental anxiety) adjusted for baseline (pretest) MDAS total and factor scores and age, taking into account the effect of blocking.

**Results:**

Total and anticipatory dental anxiety decreased more in the VRR group than the TAU group (β = −0.75, P < .001, for MDAS total score; β = −0.43, P < .001, for anticipatory anxiety score) in patients of a primary dental care clinic. In women, dental anxiety decreased more in VRR than TAU for total MDAS score (β = −1.08, P < .001) and treatment-related dental anxiety (β = −0.597, P = .011). Anticipatory dental anxiety decreased more in VRR than TAU in both men (β = −0.217, P < .026) and women (β = −0.498, P < .001).

**Conclusion:**

Short application of VRR is both feasible and effective to reduce preoperative dental anxiety in public dental care settings (ClinicalTrials.gov NCT03993080).

**Knowledge Transfer Statement:**

Dental anxiety, which is a common problem, can be reduced with short application of virtual reality relaxation applied preoperatively in the waiting room. Findings of this study indicate that it is a feasible and effective procedure to help patients with dental anxiety in normal public dental care settings.

## Introduction

One-third of Finnish adults are anxious of dental treatment to some degree, women more often than men. A tenth are very anxious. The prevalence of dental anxiety has remained stable over the past 10 y (Lahti et al. 2007; Liinavuori et al. 2016). These statistics are similar in other countries (Hägglin et al. 1999; Maggirias and Locker 2002; Thomson et al. 2009; Armfield 2010; Hill et al. 2013; Carlsson et al. 2015). People with extreme dental anxiety are more likely to avoid or delay treatment (Pohjola et al. 2007; Thomson et al. 2010; Åstrøm et al. 2011; Hakeberg and Wide Boman 2017; Liinavuori et al. 2019), Finnish men more often than women (Liinavuori et al. 2019).

Dental anxiety may be managed by psychotherapeutic interventions, which enable patients to feel more comfortable when receiving the treatment and which help those patients not visiting the dentist due to a high fear to attend the treatment. These interventions include relaxation, distraction, exposure, and other forms of cognitive behavioral therapy (Armfield and Heaton 2013; Gordon et al. 2013; Wide Boman et al. 2013; Craske et al. 2014). Of these, relaxation and distraction are mostly used during dental treatment, whereas exposure therapy, including inhibitory learning, and other forms of cognitive behavioral therapy might be needed before the dental treatment (Armfield and Heaton 2013; Craske et al. 2014). While some of these interventions may be conducted by a dentist, others require support from psychologists (Armfield and Heaton 2013; Wide Boman et al. 2013). Several treatment visits are usually needed to manage dental anxiety, especially for those with extreme dental anxiety; however, a single appointment to reduce dental anxiety has also shown some success (Armfield and Heaton 2013; Gordon et al. 2013; Wide Boman et al. 2013). Based on this research evidence, a brief patient-centered intervention is needed that may be routinely incorporated into daily practice in primary dental care. New technologies have been developed, such as computer-assisted cognitive behavioral therapy, which has shown some potential (Rooksby et al. 2015; Tellez et al. 2015). Technologies based on virtual reality have also been developed for managing dental anxiety. A systematic review concluded that they have potential, though more rigorous studies are needed (Gujjar et al. 2019a). Many of them are based on distraction during normal or simulated treatment or exposure before treatment and used, for example, natural scenery, games, or information on treatment (Frere et al. 2001; Asl Aminabadi et al. 2012; Tanja-Dijkstra et al. 2014; Kazancioglu et al. 2015; Padrino-Barrios et al. 2015; Atzori et al. 2018; Niharika et al. 2018; Shetty et al. 2019), while others are based on psychologist-delivered cognitive behavioral therapy (Raghav et al. 2016; Gujjar et al. 2017; Gujjar et al. 2019b). Short virtual reality–based interventions have shown particular promise in reducing preoperative or anticipatory anxiety in secondary care (Ganry et al. 2018). We are unaware, however, of short virtual reality–based relaxation being applied in primary dental care preoperatively.

Therefore, our research question is as follows: Can a short virtual reality–based intervention applied preoperatively be effective in reducing patients’ anticipatory and treatment-related dental anxiety for those attending primary dental care? The aim is to apply short-term virtual reality relaxation (VRR) to examine if it is effective in reducing anticipatory and treatment-related dental anxiety in primary dental care through a randomized controlled trial (RCT) design.

## Methods

### Design

A randomized controlled single-center trial was conducted with 2 parallel arms: VRR and treatment as usual (TAU). Groups were randomized, following consent, with an allocation ratio of 1:1. No changes were made to methods after trial commencement.

### Participants

Adult patients (≥18 y) who attended for dental treatment (basic, special, or emergency dental care; general anesthesia, x-ray), consented, and were able to complete the Finnish questionnaire without assistance were eligible for the study.

The study was conducted in the public Oral Health Care Unit of the Kalasatama Health and Welfare Center of the City of Helsinki, Finland. Patient recruitment and running the on-site research activities, such as administering the questionnaires and instructing the VRR group in the use of appliances, were conducted by 13 students from the Haaga-Helia University of Applied Sciences and Laurea University of Applied Sciences. Students were trained for this study by the lead clinician (S.L.) on-site to ensure uniformity of information provided to participants.

Patients were approached in 1 of the 2 arrival halls where they entered the Oral Health Care Unit. Patients were inquired if they had 15 min before their scheduled dental appointment to allow participation in the study. If the patients had the time and volunteered, they were told the nature of the study and given an information leaflet describing it and the possibility to win a movie ticket or xylitol products in a lottery after participation. If the patient consented, she or he was then randomized into 1 of the 2 groups.

### Interventions

Interventions were conducted in similar settings in small alcoves with a seat and a table. The participants in the TAU group remained seated in the alcove for 3 min. Their experience of sitting in the alcove for 3 min was identical to that of the VRR group but without the VRR intervention. They were able to use their mobile phones if they so wished.

In the VRR group, participants chose 1 of the 5 videos (1 to 3.5 min). Still pictures of each video are provided in the Appendix. The application by MelloVR presented these videos. When the application was launched, clear instructions were displayed on the screen regarding next steps. These included basic instructions on how to select a video by turning one’s head toward a specific video via the so-called gaze selection method without manual controllers. The 360° videos (resolution range, 4,096 × 2,010 to 5,120 × 2,560) immersed the participants in a peaceful virtual landscape (beach, waterfall, underwater, space float, paddling). Videos were played with a Samsung Gear VR headset and a Samsung Galaxy S7 mobile phone (attached to the virtual headset) for the MelloVR application, with a total weight of approximately 500 g. A disposable mask was used with the headset for hygienic purposes.

Audio features and sound supported the relaxation experience. The musical ambient track was the same for all video choices. The file format is AAC with 320-kbps quality playing at 48 kHz. It has a tempo of 120 bpm (beats per minute) and fades in smoothly within 10 s. The musical instrumentation consists of a smooth synth pad, soft kick drum, and occasional bass and bell notes. White noise can be heard on top of the track, which listeners might find relaxing, particularly people with tinnitus. The synth pad looped the same harmony throughout the musical track, and the bass supports it. The bell instrument can be heard a few times, but no specific theme is recognized. This is typical of musical productions that are not meant to raise significant attention. The sound was played with on-ear headphones by Pioneer (model SE-M521) to exclude noise. The picture could be adjusted to suit the user’s eyesight by using the scroll on top of the glasses, and the audio volume could be set accordingly with a control on the side of the glasses.

Acceptability and feasibility of the VRR application were pilot tested prior to the RCT in 55 primary health care and social welfare clients of the Kalasatama Health and Welfare Center. Students who later recruited participants in the RCT invited volunteering clients to try a relaxing virtual reality experience. The virtual reality content and the devices were similar to those in the study. Volunteers’ perceptions were assessed after the virtual reality experience. Of the pilot participants, 98% found the experience relaxing; 87% would like to use it during a potentially anxiety-provoking treatment procedure; and 80% would recommend it to friends. Minor harmful effects, such as feelings of dizziness or nausea, were reported by <4%.

### Outcomes

The main outcome measure, dental anxiety, was assessed with the validated Finnish version of the Modified Dental Anxiety Scale (MDAS) before and immediately after the intervention (Humphris et al. 2000; Yuan et al. 2008; Humphris et al. 2013). The measure has 5 questions, each with 5 reply alternatives from *not anxious* to *extremely anxious*. The primary outcome variable was the posttest MDAS total score. The secondary outcome variables were posttest scores for the 2 subscales of the MDAS: anticipatory dental anxiety (MDAS items 1 and 2) and treatment-related dental anxiety (MDAS items 3 to 5). After the intervention and completion of the posttest MDAS, patients reported their gender (female, male, other) and age in full years before attending their scheduled dental appointment. No personal information or information related to dental appointments after the study was collected.

From the MDAS, sums were calculated for the primary outcome total scale (range, 5 to 25) and for the secondary outcomes: anticipatory dental anxiety (range, 2 to 10) and treatment-related dental anxiety (range, 3 to 15).

### Sample Size

Power calculation was estimated by the Stata “rsquared” routine. The effect of blocking was not introduced; however, the effect size was set to a low level to ensure a conservative approach when estimating a sufficient sample size. A small effect size of 0.04 in favor of the VRR intervention as compared with TAU would require a sample size of 272 participants at 90% power with alpha set to 0.05, 2-sided. This was calculated by specifying 2 control covariates (pretest MDAS and participant age in years) and the test random assignment factor (0 = TAU, 1 = VRR). Due to the chosen block size of 10 participants, the study required 280 participants.

### Randomization

A random allocation sequence was computer generated by A.S. using random number lists in blocks of 10. The blocked randomization was used to keep the numbers of patients in both treatment groups closely balanced during the study and thus to homogenize the variation in group allocation due to patient flow in different weekdays and time of day. The block size of 10 was big enough to prevent guessing the next randomized treatment group, thus reducing bias (Altman 1991). The block size of 10 was also the multiple of number of treatments, and the required sample size was divisible by block size. The students enrolling the participants administered the randomization of patients, allocating the patient to the next free identification number on the randomization list. The patients were blinded until the intervention started. It was not possible to blind the students enrolling the patients.

### Statistical Analyses

The primary outcome variable, posttest MDAS total score, was adjusted for the baseline (pretest) MDAS total score and participant age through mixed effects regression with inclusion of the random block effect. The analysis method ignoring blocks is more conservative regarding the statistical significance and thus less efficient and powerless (Matts and Lachin 1988). The analyses were repeated for the secondary outcome variables: MDAS anticipatory and treatment-related dental anxiety. Separate analyses were run for males and females. To avoid making assumptions of strict normality and nonheteroscedasticity, the “robust” option in the “regress” procedure was applied. Residual plots were inspected for identification of possible violations. Alpha was set to 0.05 (2-sided). Data were analyzed with Stata 15.1 (StataCorp 2017).

### Ethics

Ethical approval was granted by the City of Helsinki (HEL 2018-008940). The trial was registered at ClinicalTrials.gov (NCT03993080).

## Results

The flowchart of allocated and analyzed participants is presented in the Figure. Data collection started October 15, 2018, and was completed February 27, 2019. Recruitment was halted at 277 participants, who were analyzed by original assigned groups. Means and standard deviations for age and the MDAS total, anticipatory, and treatment-related anxiety scores according to gender and intervention group are presented in Table 1. Of the participants, 47.5% reported low dental anxiety (MDAS <10); 43.9%, moderate dental anxiety (MDAS, 10 to 18); and 8.6%, high dental anxiety (MDAS ≥19).

**Figure.**
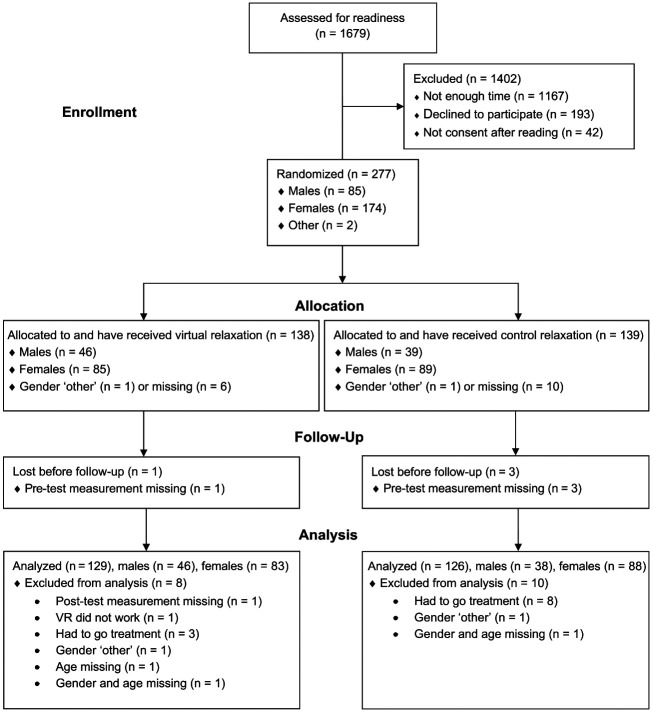
CONSORT flow diagram of allocated and analyzed participants. VR, virtual reality.

**Table 1.** Age and Dental Anxiety (Total and 2 Factors) for Males and Females and the VRR and TAU Groups.

	All (*N* = 255)	Males (*n* = 84)	Females (*n* = 171)	VRR (*n* = 129)	TAU (*n* = 126)
Age, y	52.5 (16.4)	50.2 (16.4)	53.7 (16.3)	51.8 (16.8)	53.3 (16.0)
Baseline					
MDAS total	11.0 (4.7)	9.4 (4.1)	11.8 (4.7)	10.8 (4.5)	11.2 (4.8)
Anticipatory	3.7 (2.0)	3.2 (1.7)	3.9 (2.0)	3.6 (1.9)	3.7 (2.0)
Treatment	7.3 (3.1)	6.2 (2.7)	7.9 (3.1)	7.1 (3.0)	7.5 (3.2)
After treatment					
MDAS total	10.3 (4.6)	9.2 (4.3)	10.9 (4.6)	9.8 (4.3)	10.9 (4.7)
Anticipatory	3.5 (1.9)	3.1 (1.8)	3.7 (2.0)	3.3 (1.9)	3.8 (2.0)
Treatment	6.8 (2.9)	6.1 (2.7)	7.2 (3.0)	6.5 (2.8)	7.1 (3.1)

Values are presented as mean (SD).

MDAS, Modified Dental Anxiety Scale; TAU, treatment as usual; VRR, virtual reality relaxation.

Group had a statistically significant effect in the total MDAS model and anticipatory dental anxiety model (Table 2). The VRR group showed 0.75–MDAS scale unit decrease in total dental anxiety and a 0.43–scale unit decrease in the anticipatory dental anxiety as compared with the TAU group. In the secondary gender-specific analyses, the females in the VRR group showed a >1–MDAS scale unit decrease in dental anxiety as compared with the TAU group. For males, the decrease was not statistically significant. In MDAS anticipatory dental anxiety. the VRR group showed a half–scale unit decrease as compared with the TAU group in females and a 0.2-unit decrease in males. For treatment-related dental anxiety, the decrease in MDAS scores was statistically significant only among females in the VRR group, showing over a half–scale unit decrease as compared with the TAU group (Table 3).

**Table 2.** Mixed Effects Linear Regression Model for the Posttest Dental Anxiety (Total and 2 Factors) Adjusted for Baseline MDAS Total Score, Gender, Age, and Adapting the Blocking.

	All (*N* = 255)		
	β	95% CI	*P* Value
Total MDAS after			
Group (VRR vs. TAU)	−0.752	−1.183 to −0.321	0.001
Total MDAS before	0.889	0.817 to 0.961	<0.001
Gender	−0.443	−0.933 to 0.047	0.076
Age in years	0.003	−0.011 to 0.018	0.642
Anticipatory after			
Group (VRR vs. TAU)	−0.429	−0.650 to −0.207	<0.001
Anticipatory before	0.885	0.828 to 0.942	<0.001
Gender	−0.099	−0.343 to 0.146	0.415
Age in years	−0.002	−0.008 to 0.005	0.659
Treatment after			
Group (VRR vs. TAU)	−0.338	−0.694 to 0.018	0.062
Treatment before	0.849	0.789 to 0.909	<0.001
Gender	−0.275	−0.677 to 0.127	0.171
Age in years	0.003	−0.008 to 0.014	0.573

β, nonstandardized coefficient; MDAS, Modified Dental Anxiety Scale; TAU, treatment as usual; VRR, virtual reality relaxation.

**Table 3.** Gender-Specific Mixed Effects Linear Regression Models for the Posttest Dental Anxiety (Total and 2 Factors) Adjusted for the Baseline MDAS Total and Factor Scores, Age, and Adapting the Blocking.

	Male (*n* = 84)			Female (*n* = 171)		
	β	95% CI	*P* Value	β	95% CI	*P* Value
Total MDAS after						
Group (VRR vs. TAU)	−0.123	−0.517 to 0.270	0.539	−1.084	−1.685 to −0.484	<0.001
Total MDAS before	0.999	0.933 to 1.065	<0.001	0.846	0.761 to 0.932	<0.001
Age in years	0.010	−0.005 to 0.025	0.201	−0.001	−0.018 to 0.016	0.921
Anticipatory after						
Group (VRR vs. TAU)	−0.217	−0.409 to −0.025	0.026	−0.498	−0.744 to −0.253	<0.001
Anticipatory before	0.970	0.901 to 1.039	<0.001	0.846	0.744 to 0.948	<0.001
Age in years	0.007	0.001 to 0.014	0.046	−0.006	−0.014 to 0.002	0.128
Treatment after						
Group (VRR vs. TAU)	0.091	−0.295 to 0.476	0.645	−0.597	−1.06 to −0.138	0.011
Treatment before	0.953	0.854 to 1.052	<0.001	0.815	0.734 to 0.896	<0.001
Age in years	0.002	−0.010 to 0.014	0.716	0.004	−0.009 to 0.016	0.594

β, nonstandardized coefficient; MDAS, Modified Dental Anxiety Scale; TAU, treatment as usual; VRR, virtual reality relaxation.

The MDAS outcome data showed a significant level of skewness. The “robust” option in Stata was applied to mitigate this. To check that our analyses were unbiased, we repeated the regression analyses with log-transformed dependent variable. All statistical results remained substantively the same.

## Discussion

A short preoperative VRR decreased total and anticipatory dental anxiety in those attending a primary dental care clinic. In the secondary gender-specific analyses, total and treatment-related dental anxiety decreased among females and anticipatory dental anxiety among males. To our knowledge, this is the first study with a short VRR method in a routine dental primary care setting. Like Ganry et al. (2018), we found that even a short application of VRR reduced anticipatory dental anxiety.

It is possible that at least part of the dental anxiety reduction came from distraction, which has been shown to be effective when applied during dental treatment (Frere et al. 2001; Asl Aminabadi et al. 2012; Tanja-Dijkstra et al. 2014; Padrino-Barrios et al. 2015; Atzori et al. 2018; Niharika et al. 2018; Shetty et al. 2019). The virtual reality used in this study was developed for relaxation purposes. Regardless of the pathway, the use of virtual reality preoperatively reduced dental anxiety.

The strengths of this study are the RCT design and the study population, which included participants with all levels of dental anxiety in the primary dental care setting. The levels of dental anxiety were similar to the UK population norms (Humphris et al. 2013). We did not aim to maximize the effect of VRR by recruiting participants with high levels of dental anxiety only. Also, the intervention setup was very similar for both groups in terms of seating and the possibility for the TAU group to use a mobile phone, thus enabling the effect of the virtual reality intervention to be explicitly identified. The study did not assess dental anxiety levels after dental treatment or the type of treatment procedures that participants were receiving. Neither was the content or length of the VRR intervention that participants chose assessed, as this was a population study. Thus, the long-term effects and the effects of different VRR interventions as well as different dental treatments call for further studies.

There are also limitations to the study population. Recruiting took place in a setting with on average 200 patient visits per day. However, most patients arrived just in time for their scheduled appointment and did not have sufficient time to participate in the study (69.5% of those approached and 83.2% of those excluded). This might have led to possible bias in the age distribution, as older patients were more likely to arrive ahead of their scheduled appointments and thus participate the study. As another recruitment bias, we might have missed patients with high dental anxiety, as they may have come at the last minute. However, the percentages of participants with high dental anxiety were similar to the national survey among adult Finns (Liinavuori et al. 2016) and possibly due to the recruitment including patients coming for acute dental care. Only 11.5% of those approached declined to participate for other reasons, and 2.5% did not consent after reading the written information. The fact that many patients were unable to seek out VRR treatment due to time constraints needs to be addressed to ensure successful implementation at the population level.

There was also a lower percentage of men than women in this study, with only 3 men reporting high dental fear in this study. Men with high dental fear were underrepresented in another cohort study where dental anxiety was assessed in conjunction with dental examination (Kankaanpää et al. 2019). This might partly explain the lack of statistical significance of VRR among men and needs to be considered when powering future studies. Thus, results referring to the effect of gender should be interpreted with caution.

The positive findings of this study indicate that a short VRR intervention is a feasible, patient-accepted, inexpensive, and effective way of reducing preoperative dental anxiety in a public dental care setting on a population level. For those who are truly dentally phobic, we realize that more in-depth psychotherapeutic interventions are necessary. We therefore recommend in future studies that the level of dental anxiety be carefully inspected. In addition, further studies are needed to understand the effect of this VRR intervention more fully and to assess long-term outcomes.

## Author Contributions

S. Lahti, G. Humphris, contributed to conception, design, data analysis, and interpretation, drafted and critically revised the manuscript; A. Suominen, contributed to design, data analysis, and interpretation, drafted and critically revised the manuscript; R. Freeman, contributed to conception, design, and data interpretation, drafted and critically revised the manuscript; T. Lähteenoja, contributed to design and data interpretation, drafted and critically revised the manuscript. All authors gave final approval and agree to be accountable for all aspects of the work.

## Supplemental Material

DS_10.1177_2380084420901679 – Supplemental material for Virtual Reality Relaxation to Decrease Dental Anxiety: Immediate Effect Randomized Clinical Trial Click here for additional data file. Supplemental material, DS_10.1177_2380084420901679 for Virtual Reality Relaxation to Decrease Dental Anxiety: Immediate Effect Randomized Clinical Trial by S. Lahti, A. Suominen, R. Freeman, T. Lähteenoja and G. Humphris in JDR Clinical & Translational Research
