# Supplementary material for: Virtual Reality Relaxation to Decrease Dental Anxiety: Immediate Effect Randomized Clinical Trial
Source: JDR Clin Trans Res. 2020 Jan 21;5(4):312–8. doi: 10.1177/2380084420901679 (PMC7495687; doi:10.1177/2380084420901679)
Supplement: DS_10.1177_2380084420901679 – Supplemental material for Virtual Reality Relaxation to Decrease Dental Anxiety: Immediate Effect Randomized Clinical Trial [file DS_10.1177_2380084420901679.pdf]

Virtual Reality Relaxation to Decrease Dental Anxiety: Immediate Effect Randomized Clinical Trial

S. Lahti, A. Suominen, R. Freeman, T. Lähteenoja, and G. Humphris

Appendix

Still pictures of the five videos used in the virtual reality relaxation intervention

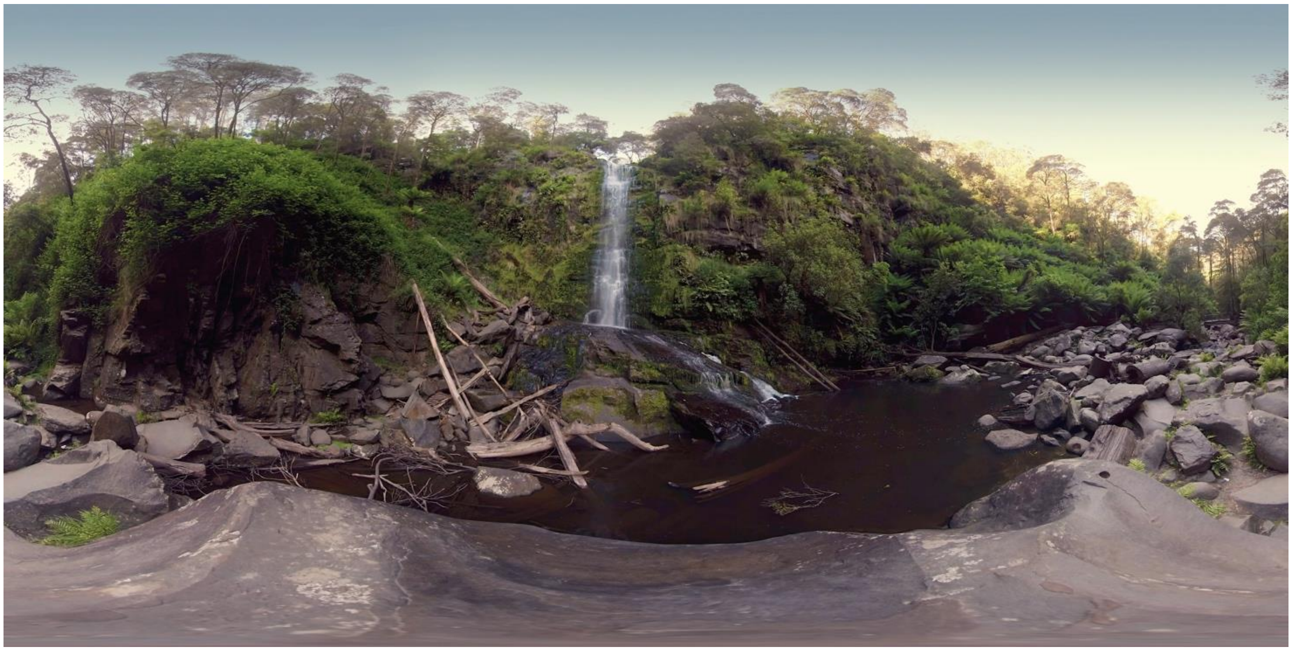

Video 1. Australian Waterfalls

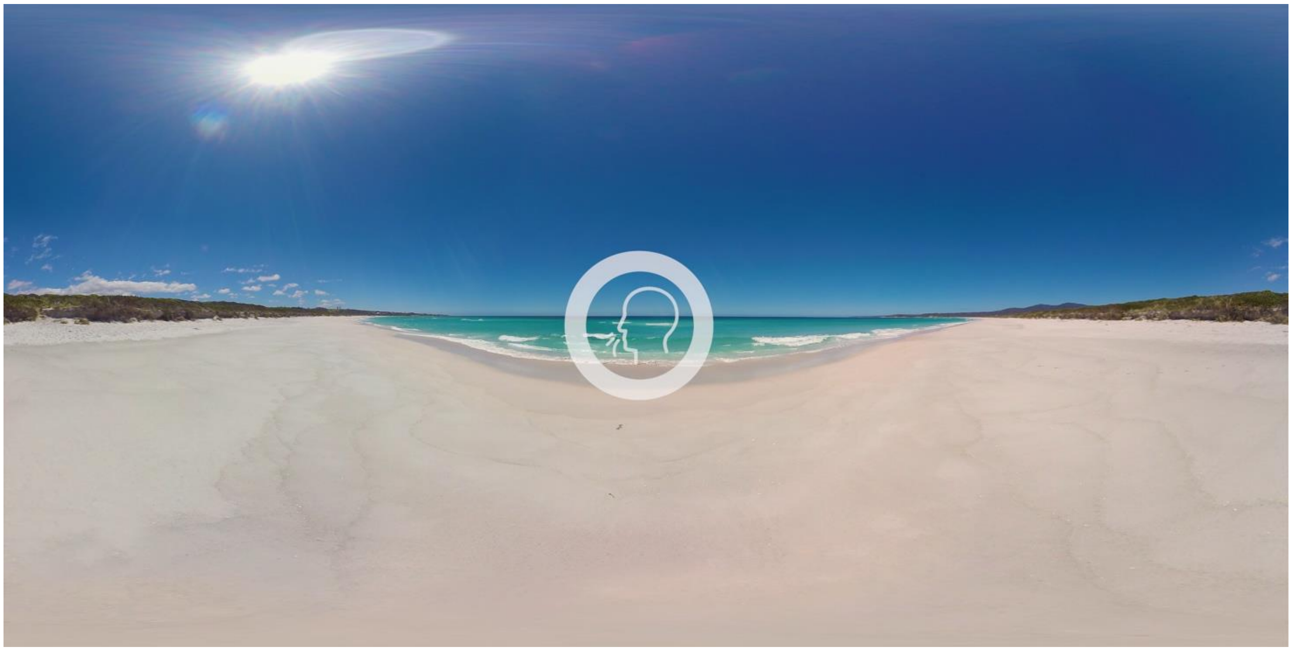

Video 2 Breathing Exercise on Philippine Beach

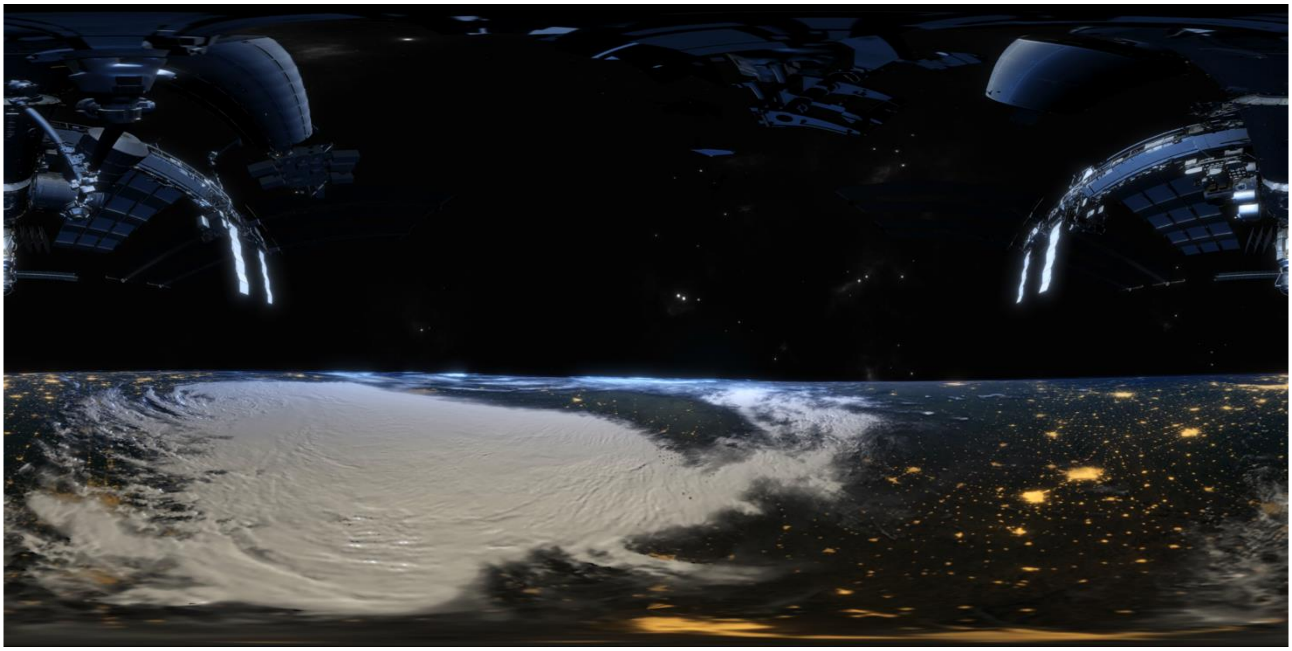

Video 3. Floating in Space

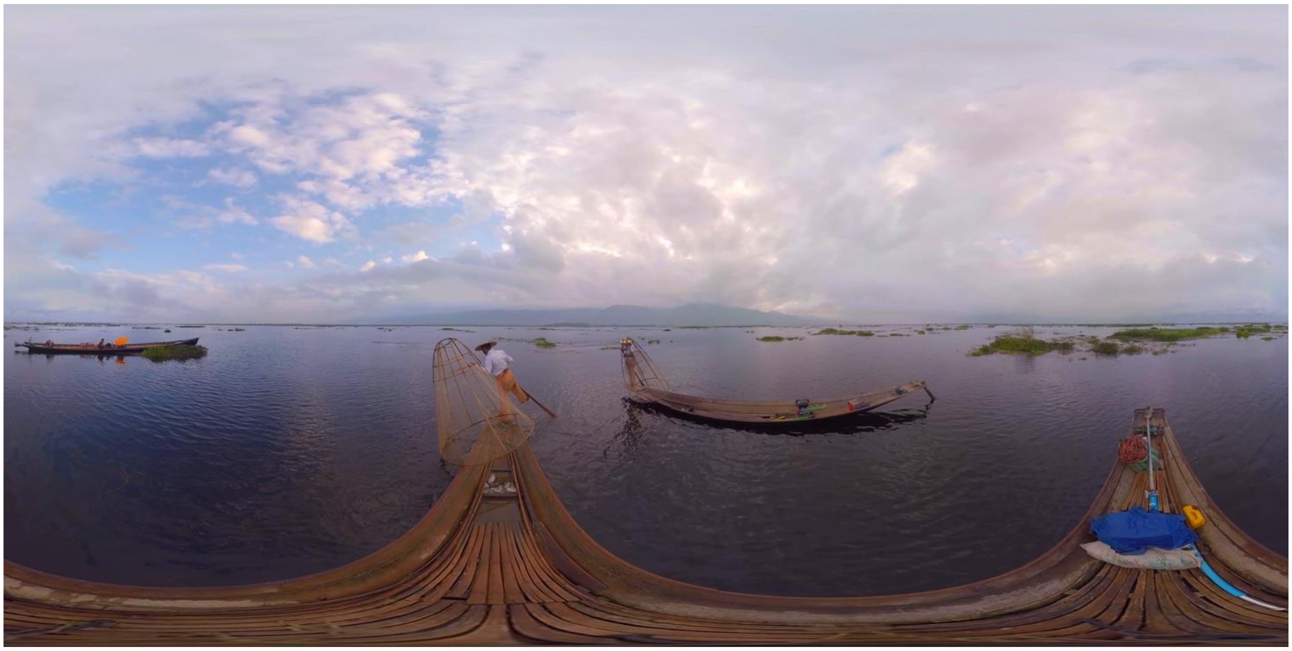

Video 4. Island Hopping in Thailand

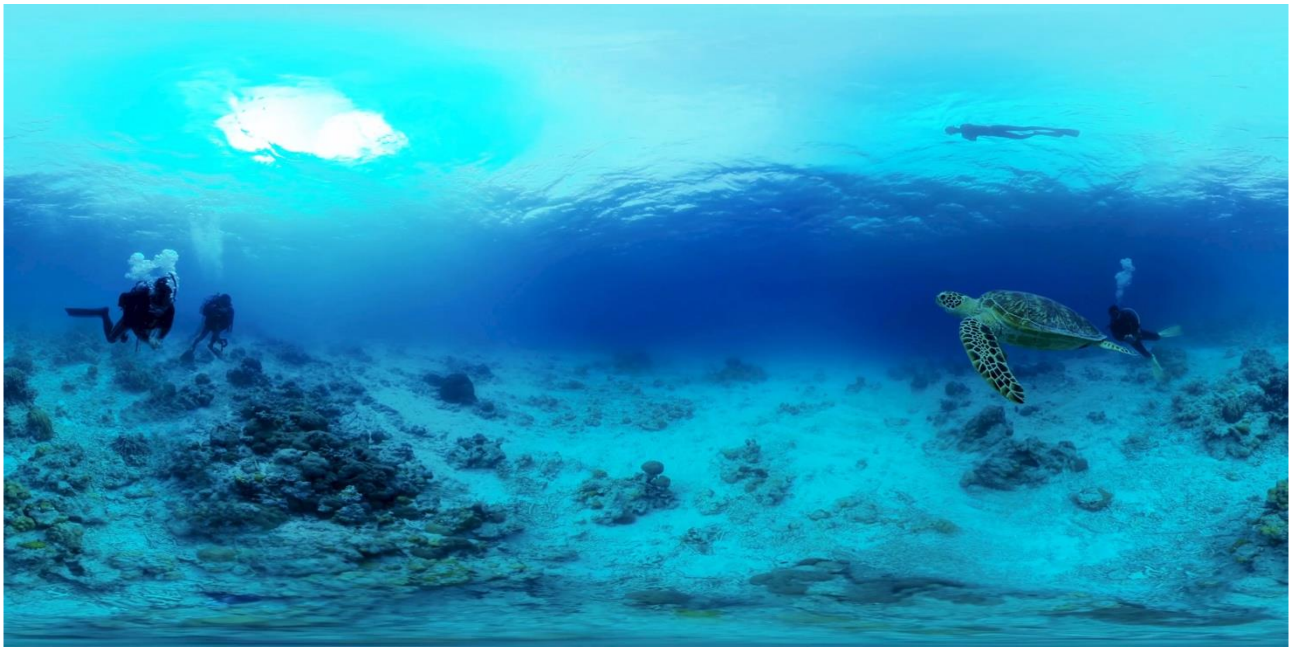

Video 5. Turtle Dive in Bora Bora
